# Complete Genome Sequence for *Asinibacterium* sp. Strain OR53 and Draft Genome Sequence for *Asinibacterium* sp. Strain OR43, Two Bacteria Tolerant to Uranium

**DOI:** 10.1128/MRA.01701-18

**Published:** 2019-04-04

**Authors:** Ryann M. Brzoska, Marcel Huntemann, Alicia Clum, Amy Chen, Nikos Kyrpides, Krishnaveni Palaniappan, Natalia Ivanova, Natalia Mikhailova, Galina Ovchinnikova, Neha Varghese, Supratim Mukherjee, T. B. K. Reddy, Chris Daum, Nicole Shapiro, Tanja Woyke, Annette Bollmann

**Affiliations:** a Department of Microbiology, Miami University, Oxford, Ohio, USA; b DOE Joint Genome Institute, Walnut Creek, California, USA; University of California, Riverside

## Abstract

*Asinibacterium* sp. strains OR43 and OR53 belong to the phylum *Bacteroidetes* and were isolated from subsurface sediments in Oak Ridge, TN. Both strains grow at elevated levels of heavy metals. Here, we present the closed genome sequence of *Asinibacterium* sp. strain OR53 and the draft genome sequence of *Asinibacterium* sp. strain OR43.

## ANNOUNCEMENT

*Asinibacterium* sp. strains OR43 and OR53 (formerly *Sediminibacterium* sp. strains OR43 and OR53) are Gram-negative, nonmotile, aerobic bacteria. The type strain *Asinibacterium lactis* was isolated from donkey milk powder (1). Closely related genera include *Sediminibacterium*, *Vibrionimonas*, and *Hydrotalea* (2–6). Related sequences (16S rRNA) were detected ubiquitously in the environment but most notably in sites contaminated with hydrocarbons, heavy metals, and/or radionucleotides (7–14). The genome sequences will provide insight into the potential role of *Asinibacterium* sp. strains OR43 and OR53 in the bioremediation of heavy metals.

*Asinibacterium* sp. strains OR43 and OR53 were isolated from the contaminated subsurface sediment at the Integrated Field Research Challenge (IFRC) in Oak Ridge, TN, with the diffusion chamber approach (7). Both strains have a very similar physiology and are able to grow in the presence of uranium equal to the concentrations in their original environment (7, 15) (R. M. Brzoska and A. Bollmann, unpublished data). Prior to DNA isolation, the strains were grown in 0.1× Luria-Bertani broth at 27°C (7). Genomic DNA was isolated with the JETFLEX genomic DNA purification kit from GenoMed (Loehne, Germany) according to the manufacturer’s recommendations. Genome sequence data for both genomes were obtained with the Illumina HiSeq 2000 platform with paired-end technology (2 × 150 bp) (16). The data produced 18,342,342 reads generating 3,005 Mbp (strain OR43) and 21,794,720 reads generating 3,269 Mbp (strain OR53). The genome sequences were assembled with ALLPATHS version R37654 (strain OR43) and version R39750 (strain OR53) (17), Velvet version 1.1.05 (18), and Phrap version 4.24 (High Performance Software LLC) (only strain OR53). Prodigal 2.5 was used for gene calling (19). The genomes were annotated with the DOE Joint Genome Institute (JGI) Annotation Pipeline (20, 21) and further analyzed with the Integrated Microbial Genomes and Microbiomes database and comparative analysis system (IMG/M) at the Joint Genome Institute in Walnut Creek, CA (22).

The genome size for *Asinibacterium* sp. strain OR43 was 3,768,016 bp in 12 scaffolds with a GC content of 45.7%, and that for *Asinibacterium* sp. strain OR53 was 3,715,967 bp in 1 scaffold with a GC content of 45.4%. *Asinibacterium* sp. strain OR43 had 2,473 proteins with predicted functions out of 3,284 protein-coding sequences, while *Asinibacterium* sp. strain OR53 had 2,464 proteins with predicted functions out of 3,281 protein-coding sequences. The genomes contained predicted heavy-metal efflux pumps and sensing proteins. The average nucleotide identity (ANI) between the genomes was calculated with the Microbial Species Identifier (MiSI) method (23) at 96.4%, which indicated that the genomes were the same species and very closely related. Further analysis is needed to determine the mechanism of *Asinibacterium* spp. to withstand and grow in the presence of uranium.

### Data availability.

This whole-genome project has been deposited at DDBJ/EMBL/GenBank under the accession numbers ATYE00000000 (*Asinibacterium* sp. strain OR43) and AZXP00000000 (*Asinibacterium* sp. strain OR53). The raw reads were deposited in the SRA under SRP078705 (*Asinibacterium* sp. strain OR53) and SRP078706 (*Asinibacterium* sp. strain OR43).
